# Acceptance and use of digital health technologies among physiotherapists in Germany: a web-based cross-sectional survey

**DOI:** 10.1186/s12913-026-14005-3

**Published:** 2026-01-10

**Authors:** Fatma Sahan, Anja Gutermuth, Jennifer A. Müller, Thomas Muth, Karin Panitz, Jennifer Apolinário-Hagen

**Affiliations:** 1 Institute for Occupational, Social and Environmental Medicine and Center for Digital Medicine, Medical Faculty, University Hospital Düsseldorf, Heinrich-Heine- University Düsseldorf, Düsseldorf, Germany; 2 Institute for Occupational, Social and Environmental Medicine, Medical Faculty, University Hospital Düsseldorf, Heinrich-Heine-University Düsseldorf, Düsseldorf, Germany

**Keywords:** Physiotherapists, Physical therapists, Physical therapy modalities, Digital health, Telemedicine, Technology acceptance, Mobile applications, Virtual reality

## Abstract

**Background:**

Digital health technologies (DHT) have the potential to improve physiotherapy efficiency, access, and patient engagement, yet their uptake among physiotherapists remains limited. However, research into the individual determinants of DHT acceptance and professional use in physiotherapy practice remains scarce in Germany.

**Objectives:**

This study investigates the acceptance of DHT among physiotherapists in Germany, identifying key factors influencing acceptance and actual use. Individual determinants such as demographic characteristics, eHealth literacy, technology readiness, and acceptance factors based on the Unified Theory of Acceptance and Use of Technology (UTAUT) model are considered.

**Methods:**

A cross-sectional online survey of physiotherapists in Germany was conducted from November to December 2023. The questionnaire assessed UTAUT scales, including performance expectancy (PE), effort expectancy (EE), social influence (SI), and facilitating conditions (FC), as well as eHealth literacy, technology readiness, demographic characteristics, knowledge about DHT, and exposure to digital health in formal education. Multiple linear regression analyses were performed using R.

**Results:**

Data from 297 physiotherapists (*M* = 44.56, *SD* = 12.26, female = 72.23%) were analyzed. Multiple regression analyses revealed that the UTAUT scales PE, FC, and SI, as well as the additional moderator formal education in digital health, were significantly associated with higher DHT acceptance (explained variance: *R*² = 0.79). Older age and prior DHT use negatively influenced acceptance, while knowledge, technology readiness, and eHealth literacy showed no significant influence. Younger physiotherapists used innovative digital tools (sensors: *p* = .002; wearables: *p* = .002; web apps: *p* = .008) more frequently. Male physiotherapists reported higher DHT acceptance than female professionals (*p* = .020). Notably, physiotherapists who had never used DHT showed greater acceptance than prior users (*p* < .001).

**Conclusions:**

Acceptance of DHT is significantly shaped by key UTAUT constructs, notably PE, SI, and FC, with PE identified as the most influential determinant. Age-related differences were evident, as younger physiotherapists reported higher levels of digital tool acceptance. Training tailored to individual needs and technical support could enhance acceptance across age groups. Future research should investigate specific barriers to integration to help develop practical strategies for incorporating DHT in physiotherapy.

## Background

Physiotherapy is a health profession that focuses on maintaining, promoting, and restoring the body’s mobility and functionality [1]. The approach incorporates active motor exercises, manual techniques and physical applications with the objective of alleviating pain, mitigating movement disorders, and enhancing quality of life [1, 2]. Physiotherapeutic care is characterized by its active engagement with patients, encompassing promotion, prevention, treatment, and rehabilitation in various clinical contexts [2]. By addressing functional limitations that affect daily activities, participation, and independence, physiotherapy contributes to reducing the global burden of disease associated with impaired movement and physical function. Musculoskeletal disorders (MSDs) represent one prominent example, affecting an estimated 1.71 billion people worldwide and constituting a leading cause of disability [3]. Back pain alone affected 619 million people globally in 2020, with projections indicating an increase to 843 million cases by 2050 due to population growth and demographic aging [4].

Beyond the substantial disease burden, demand for physiotherapy services continues to rise across healthcare systems. Physiotherapy has been shown to provide cost-effective treatment for a broad range of conditions. In Germany, utilization of physiotherapy services among individuals with statutory health insurance increased by 1.2% in 2022, corresponding to approximately 37 million prescribed services and 257 million treatment sessions [5]. At the same time, a lack of qualified professionals is further widening the gap between demand and available services. A shortfall of more than 50,000 physiotherapists is projected by 2026 [6], which is already reflected in prolonged waiting times of 30 to 50 days before therapy initiation [7]. In Germany, most physiotherapists work in outpatient practices [8], where this shortage is particularly pronounced.

In this context, the digital transformation of healthcare can offer innovative approaches to the diagnosis, prevention, treatment, and rehabilitation across a wide range of physiotherapy-amenable conditions [9]. These include not only MSDs, but also neurological conditions (e.g., stroke or multiple sclerosis [10, 11]), cardiopulmonary diseases [12], chronic pain [13], geriatric conditions [14], and post-operative rehabilitation [9]. However, the integration of digital technologies into healthcare varies considerably across different medical fields. While disciplines such as psychiatry, gastroenterology, and endocrinology show relatively high adoption rates, the diffusion of digital health solutions appears slower and less coordinated in areas with predominantly physical, exercise-based treatment methods such as orthopedics, neurological rehabilitation, and physiotherapy more broadly [10, 15–19]. Physiotherapy across various indication areas could benefit from flexible digital therapy support (e.g., remote monitoring, digital exercise guidance, or tele-rehabilitation), which may help ease workload pressure, improve accessibility, and enhance treatment sustainability [20].

Digital health technologies (DHT) refer to a broad range of digital tools and systems used in healthcare, including telemedicine, mobile health applications, wearables, virtual reality (VR) applications, artificial intelligence (AI)-based systems such as decision-support or recommendation tools, and digital organizational infrastructures such as electronic health records (EHR), practice management software, and digital billing systems [21–23]. These technologies have the potential to facilitate the daily work of physiotherapists and increase their efficiency [24].

Beyond theoretical considerations, preliminary evidence suggests that DHT can support physiotherapy practice in various ways. For instance, DHT such as telemedicine have been shown to enable reliable remote physiotherapeutic assessments, including assessments of the knee function [24, 25]. DHT have also been demonstrated to be a cost-effective means of reaching a large number of patients while improving accessibility, particularly for patients in rural areas [26–28]. In addition to access-related benefits, DHT may provide further advantages relevant to physiotherapy, including fostering active patient engagement, enhancing health literacy, and strengthening self-efficacy [29, 30]. Moreover, DHT can facilitate the time-efficient and flexible development of personalized training programs for patients [31, 32], contribute to improved adherence to home exercise regimens [30, 33] and support increases in physical activity levels [34]. Furthermore, studies have demonstrated that rehabilitation outcomes in terms of pain, functioning, and quality of life with DHT are comparable to or even superior to traditional approaches [35–37]. These improvements may contribute to better therapeutic outcomes in physiotherapy and, in some cases, even reduce the need for surgical interventions [30, 38].

Digital health applications (German *Digitale Gesundheitsanwendungen*; DiGA) are among the key innovations in healthcare in Germany. As CE-certified medical devices of low-risk class, they are regulated by the Federal Institute for Drugs and Medical Devices (German *Bundesinstitut für Arzneimittel und Medizinprodukte*; BfArM) and listed in the official DiGA directory. As so-called “apps on prescription”, DiGA support patients with defined medical diagnoses in monitoring, treating, and alleviating diseases or impairments, primarily through self-guided digital interventions [39]. These digital therapeutics (DTx) may be provided as native apps or web-based applications (web apps), offered with or without additional devices such as wearables, and are typically used independently by patients outside of clinical encounters, with optional support of communication with healthcare providers [40]. With the introduction of DiGA, Germany has taken on a pioneering role in the digital transformation of healthcare [41]. Several DiGA are already available for physiotherapeutic conditions. As of December 8, 2025, seven of the 57 DiGA listed in the directory of the BfArM [42] are categorized under “muscles, bones, and joints”. One of these applications focuses on chronic pain management, two address knee disorders, another three are designed for back pain, and one specifically targets shoulder lesions [42]. In the DiGA directory, the category entitled “Nervous system” includes an additional DiGA relevant to physiotherapy, for example for multiple sclerosis. While physiotherapists in Germany, unlike physicians and psychotherapists, are not legally allowed to diagnose illnesses or prescribe DiGA, they can support patients in using DiGA and integrating them into daily life (e.g., home exercises).

Despite the promising possibilities of digital transformation, research indicates that acceptance and use of DHT remain relatively low, potentially reflecting reservations among physiotherapists toward these technologies [43, 44]. Barriers to the acceptance and use of DHT among physiotherapists include data protection concerns, lack of technical infrastructure, low perceived usefulness for their own work or practice, and insufficient digital competencies [44, 45].

The physiotherapy sector needs effective strategies to integrate digital technologies into daily practice, particularly through competence development programs. Not only in Germany but also internationally, “Digital Health” has yet to be systematically incorporated into the education and training of physiotherapists [22]. There is a particular lack of robust data on the acceptance of DHT among physiotherapists in Germany, regardless of their user experience or familiarity with specific applications (e.g [44]). Likewise, little is known about the individual determinants influencing the acceptance of DHT among physiotherapists, particularly in the German healthcare context.

### Objective of the present study

The objective of this study is to examine the acceptance of DHT, particularly in their application for therapy support, within the field of physiotherapy in Germany. The study aims to identify key factors influencing the acceptance (in terms of intention to use) and actual use of DHT by physiotherapists. A variety of individual determinants are considered, including personal views (beliefs or expectancies and attitudes), demographic characteristics, technology readiness, and self-assessed eHealth literacy and digital skills. The study seeks to gain a well-founded, context-sensitive understanding of the facilitating and inhibiting factors for the acceptance and use of DHT.

## Methods

This cross-sectional study was conducted as an online survey over eight weeks, from November 1 to December 31, 2023. Prior to data collection, the study received ethical approval from the Ethics Committee of the Medical Faculty at Heinrich Heine University Düsseldorf (ref. no.: 2023–2426).

Recruitment was carried out by AG, who holds degrees in physiotherapy and public health, across Germany through communication channels of physiotherapy professional associations (i.e., the *German Association for Physiotherapy* [Deutscher Verband für Physiotherapie], the *Association for Physiotherapy* [Verband für Physiotherapie], and the *Federal Association of Self-Employed Physiotherapists* [Bundesverband selbstständiger Physiotherapeuten]), social media platforms (e.g., Facebook, LinkedIn), informal professional networks, and printed flyers distributed in physiotherapy practices in North Rhine-Westphalia, Germany. A link to the survey software Tivian [46] allowed physiotherapists in Germany to participate after they had explicitly agreed to data processing for research purposes (“click to agree”). Before providing informed consent, participants received information on the first page of the web-based survey regarding the scope of the study, the voluntary nature of participation, and the procedures for ensuring anonymity and confidentiality. No personally identifiable data were collected, and responses could not be linked to individual participants. No monetary incentive was provided for participation.

### Development of the questionnaire and measurement instruments for acceptance and its determinants

A German-language questionnaire was developed by a physiotherapist (AG) in collaboration with the research team. Acceptance and its determinants were assessed using an adapted version of the *Unified Theory of Acceptance and Use of Technology* (UTAUT; [47]), based on previously validated adaptations by Hennemann et al. [48] and Liu et al. [49]. These adaptations have been applied in physiotherapy-related contexts. Further instruments included the German version of the *eHealth Literacy Scale* (G-eHEALS; [50]) and the *Short Scale for Measuring Technological Readiness* [51], alongside sociodemographic questions. The items for the survey targeting self-reported prior knowledge about DHT, actual use of DHT, and formal education (incl. digital health topics) were self-constructed.

A pretest of the online questionnaire was conducted with 14 participants (eight physiotherapists and six from other fields) to assess its plausibility, usability, and technical functionality. The average completion time was documented to establish criteria for later data cleaning, with excessively short completion times serving as an exclusion criterion during data analysis.

### Adapted questionnaire based on the unified theory of acceptance and use of technology

The UTAUT was developed to explain and predict technology acceptance and use [47] and integrates several established models. It comprises four core constructs: (I) *performance expectancy* (PE), defined as the extent to which using technology is expected to improve work performance; (II) *effort expectancy* (EE), referring to the perceived ease of using the technology; (III) *social influence* (SI), describing the perceived influence of the social environment on the intention to use technology; and (IV) *facilitating conditions* (FC), reflecting the perceived availability of organizational and technical support.

Within the UTAUT framework, behavioral *intention to use* technology (i.e., acceptance) is regarded as the primary predictor of actual use and is mainly influenced by PE, EE, and SI, whereas FC primarily affects use behavior [47]. The originally proposed moderating variables (age, gender, experience, and voluntariness) have shown inconsistent effects across different technological contexts [52]. In the field of digital health, FC has also been identified as a direct determinant of behavioral intention [52], while PE is widely considered the strongest determinant of technology acceptance across domains, including digital health [53].

The adapted UTAUT questionnaire comprised items assessing PE (five items), EE and SI (three items each), and FC (two items). Acceptance was operationalized as *intention to use* and measured using four items. An overall acceptance score was calculated as the mean of these four UTAUT items. All responses were recorded on a five-point Likert scale (1 = *strongly disagree*, 2 = *rather disagree*, 3 = *neither agree nor disagree*, 4 = *rather agree*, and 5 = *strongly agree*).

### German eHealth literacy scale

eHealth literacy was measured using the G-eHEALS. Originally developed by Norman and Skinner [54], this self-report instrument assesses individuals’ perceived ability and knowledge to handle health-related information in a digital context. In this study, we used the validated German version of the *eHealth Literacy Scale* [50], which consists of eight items. Responses were recorded on a five-point Likert scale ranging from 1 (*strongly disagree*) to 5 (*strongly agree*), with higher scores indicating greater eHealth literacy. The internal consistency of the scale was excellent (α = 0.91).

### Short scale for measuring technological readiness

Individual readiness to use technology was assessed using the validated *Short Scale for Measuring Technological Readiness*, which includes 12 items based on a theoretical model of technological readiness [51]. This model identifies three key dimensions that determine individual differences in technology adoption: technological acceptance (positive attitude toward technology), technological competence (perceived ability to use technology effectively), and technological control beliefs (confidence in controlling technological systems). The goal of the scale is to assess *technological readiness*, particularly in the context of adopting new technologies [51]. Responses were recorded on a five-point Likert scale ranging from 1 (*strongly disagree*) to 5 (*strongly agree*), with higher scores indicating greater technological readiness. The internal consistency of the scale in the present study was questionable (α = 0.65).

### Knowledge about digital health technologies

Respondents’ prior knowledge of DHT was assessed using an introductory set of items addressing their previous experience with digital health solutions designed to support therapeutic processes. The items were derived from an adapted questionnaire developed by Ebert et al. [55], which has previously been employed in a related context (e.g., *“I can anticipate the experience of utilizing digital health solutions for therapeutic purposes”*). In addition, two self-constructed items were included (*“I can imagine what to expect when using digital health solutions as a therapeutic tool”* and “*I already have knowledge about digital health solutions for therapy support.”*). Responses were recorded on a five-point Likert scale ranging from 1 (*strongly disagree*) to 5 (*strongly agree*), with higher scores indicating greater prior knowledge and lower scores indicating less knowledge.

### Structure of the questionnaire

The final questionnaire consisted of 53 items divided into seven sections to assess the acceptance of DHT among physiotherapists.

The first section provided participant information. The second section of the questionnaire assessed attitudes toward digitalization in physiotherapy as well as formal education in digital health. Attitudes toward the future importance of digitalization for therapy and healthcare were measured using a custom-developed item (“*How important do you think digitalization will be for therapy and healthcare in the future?*”). Responses were recorded on a five-point ordinal scale ranging from 1 (*not important at all*) to 5 (*very important*), with an additional response option “do not know/no answer“. Formal education in digital health during academic or professional training was assessed using a second custom-developed item (“*How much content on ‘digitalization’/‘e-health’ was included in your training or studies?”*), rated on a six-point ordinal scale ranging from “very many” to “none,” with higher values indicating a greater extent of digital health–related content. In addition, this section captured digital health solutions already implemented in clinical practice, including options such as EHR, telemedicine services (e.g., video therapy), practice websites, electronic appointment scheduling, social media, digital practice management or accounting software, digital anamnesis forms, and integration into telematics infrastructure. The third section included the *Short Scale for Measuring Technology Readiness*. The fourth section assessed experiences with the use of DHT in professional settings. In this section, the survey also inquired about the digital tools currently used by the surveyed physiotherapists in their practice (*“Which of the following digital health services do you already use in your daily work as part of your therapy*,* or with which have you gained experience in the past?*”). In a multiple-choice format, respondents could specify whether they had experience with online courses, sensors, smartphone apps, video consultations, VR, web apps, or wearables. Sections five and six comprised the UTAUT and G-eHEALS scales. The final section of the questionnaire collected sociodemographic data, including age, gender, professional experience, weekly working hours, type of work setting (e.g., hospital, private practice, rehabilitation center, social institution, or other), and employment status (e.g., self-employed, salaried employee, freelancer, or other).

The German questionnaire can be provided on request. The average completion time was 10 to 15 min.

### Statistical analysis

Statistical analyses were conducted using the R programming language [56], with data processing performed using the “dplyr” package [57] in version 1.0.10. Graphs were created using the “ggplot2” package, version 3.4.2 [58]. A multiple linear regression analysis was performed to examine determinants of DHT acceptance among physiotherapists. Different models were compared using adjusted *R*² as the criterion for model fit, with higher values indicating a greater proportion of explained variance in acceptance. For metric variables, mean scores were calculated. Group differences were assessed using analyses of variance (ANOVA) and non-parametric tests (Mann-Whitney U tests, Kruskal-Wallis tests), depending on data distribution and homogeneity of variances. Associations between continuous variables were examined using Pearson correlation analyses. Age groups were compared by dividing the sample into five categories: “up to 30”, “31–40”, “41–50”, “51–60”, and “over 60”. Comparisons between multiple age groups with small sample sizes were tested using Fisher’s Exact Test. Assessment of normality was based on Shapiro-Wilk tests together with visual inspection of the distributions (histograms, Q-Q plots, density curves), as recommended for robust evaluation of distributional assumptions. Homogeneity of variances was examined using Levene’s test. When assumptions were not met, non-parametric tests (Mann-Whitney U tests or Fisher’s exact tests) were applied. Post-hoc analyses were conducted using the Tukey HSD test. The statistical significance level for all tests was set at alpha < 0.05. Only fully completed datasets were incorporated into the analysis. Participants who required less than seven minutes to complete the questionnaire were excluded from subsequent data analyses.

## Results

### Sample characteristics

Out of 356 participants with completed surveys, 59 (16.57%) data sets were excluded from the analysis. The exclusion criteria included incomplete questionnaires (*n* = 9, 2.53%), completion times below the predefined threshold of seven minutes (*n* = 28, 7.87%), and conspicuous response patterns showing a strong tendency towards a single answer option (*n* = 22, 6.18%).

The final dataset comprised responses from a sample of *N* = 297 surveyed physiotherapists. Most of the participants were female (72.23%). Further descriptive data can be found in Table 1. A significant proportion of participants (*n* = 200, 67.34%) reported that digitalization content was not part of their training or studies. At the same time, 248 physiotherapists (83.50%) believed that digitalization will be (very) important for therapy and healthcare in the future.

**Table 1:** Descriptive statistics of the study participants by gender

	Total	Women	Men	Min.-Max
Participants, *n* (%)	297 (100)	216 (72.23)*	80 (26.94)*	NA
Mean age (*SD*)	44.56 (12.26)	45.38 (12.04)	42.12 (12.51)	21.00–76.00
Work experience in years (*SD*)	20.22* (12.19)	21.06 (12.23)	17.73 (11.68)	1–50
Average working hours per week (*SD*)	35.45* (10.43)	33.71 (10.18)	40.21 (9.67)	4–70

Note. Gender information was missing for a single participant. * Missing data

Most participants (73.74%) worked in private practice settings, and approximately half (47.14%) reported using at least one form of DHT, with wearables being the most prevalent type (29.29%). Practice websites (71.04%) were predominantly employed as an area of application by most participants. Descriptive analyses revealed that male physiotherapists exhibited a tendency to engage with a broader array of application areas in their practical work compared to their female counterparts. Descriptive data concerning the utilization of DHT and their domains of application in routine working life are enumerated in Table 2.

**Table 2:** Use of digital health technologies and their various applications in the daily practice of physiotherapists by gender (*N* = 297)

	Total (%)	Women (%)	Men (%)
**Work setting**			
Hospital	29 (9.76)	23 (10.65)	6 (7.50)
Private practice	219 (73.74)	156 (72.22)	63 (78.75)
Rehabilitation center	11 (3.70)	8 (3.70)	3 (3.75)
Social institution	6 (2.02)	5 (2.31)	1 (1.25)
Other	32 (10.77)	24 (11.11)	7 (8.75)*
**Use of DHT tools in daily practice**			
No use	157 (52.86)	110 (50.93)	47 (58.75)
Online courses	59 (19.87)	51 (23.61)	8 (10.00)
Sensors	57 (19.19)	37 (17.13)	20 (25.00)
Smartphone apps	63 (21.21)	37 (17.13)	26 (32.50)
Video consultation	39 (13.13)	30 (13.89)	9 (11.25)
Virtual reality	6 (2.02)	2 (0.93)	4 (5.00)
Wearables	87 (29.29)	62 (28.70)	35 (31.25)
Web apps	80 (26.94)	52 (24.07)	28 (35.00)
**Areas of application of DHT in daily practice**			
Accounting software	157 (52.86)	104 (48.15)	53 (66.25)
Digital anamnesis via email	52 (17.51)	27 (12.5)	25 (31.25)
Electronic health records	139 (46.80)	99 (45.83)	40 (50.00)
Electronic appointment scheduling	52 (17.51)	36 (16.67)	16 (20.00)
Practice management	192 (64.65)	131 (60.65)	61 (76.25)
Social media	110 (37.04)	73 (33.80)	37 (46.25)
Telematics infrastructure	10 (3.37)	5 (2.31)	5 (6.25)
Telemedicine services	34 (11.45)	25 (11.57)	9 (11.25)
Practice websites	211 (71.04)	150 (69.44)	61 (76.25)

Note. * Missing data. Multiple answers were possible. Abbreviations: DHT = digital health technologies

### Determinants of acceptance of digital health technologies for therapy support among physiotherapists based on an extended UTAUT model (multiple regression analysis and moderation models)

The model with the best fit included the variables PE, EE, SI, FC, age, eHealth literacy, formal education in digital health, knowledge, technology readiness, and actual use. The model explained 79.35% of the variance in acceptance (adjusted *R*² = 0.79, *F*(8, 288) = 109.9, *p* < .001, *AIC* = 379.78, *BIC* = 424.11). Multicollinearity diagnostics indicated that all variance inflation factor values (1.22–2.60) were well below the commonly recommended cutoff of 5.

As shown in Table 3, the UTAUT predictors PE, SI, and FC, as well as the extended determinant formal education in digital health, had a significant positive influence on acceptance. In contrast, age and actual use negatively impacted acceptance, whereas eHealth literacy, knowledge, and technology readiness showed no significant effects.

**Table 3:** Results of the linear multiple regression analysis on the determinants of acceptance (intention to use) of digital health technologies for professional use among physiotherapists (*N* = 297)

Coefficients	β	SE	t	*p*
Intercept	0.51	0.33	1.58	0.115
**UTAUT determinants**				
PE	0.40	0.04	9.98	**< 0.001**
EE	0.06	0.04	1.57	0.117
SI	0.18	0.05	3.91	**< 0.001**
FC	0.22	0.04	5.53	**< 0.001**
**Extended determinants**				
Age	− 0.01	0.003	-3.15	**0.002**
eHealth literacy	-0.05	0.05	-1.06	0.289
Formal education in digital health	0.07	0.03	2.17	**0.031**
Knowledge	0.06	0.04	1.61	0.108
Technology readiness	0.06	0.06	1.01	0.315
Actual use	-0.17	0.06	-2.60	**0.010**

Note. The predictors PE, EE, SI and FC are variables of the Unified Theory Acceptance and Use of Technology. Knowledge, technology readiness and eHealth literacy were determined using different scales. Age, formal education in digital health, knowledge, and actual use were measured using self-constructed items. Significant determinants (*p* < .05) are highlighted in bold text. Abbreviations: UTAUT = Unified Theory of Acceptance and Use of Technology; PE = Performance expectancy; EE = Effort expectancy; SI = Social influence; FC = Facilitating conditions

### Influence of age on the acceptance of digital health technologies

**Fig. 1:**
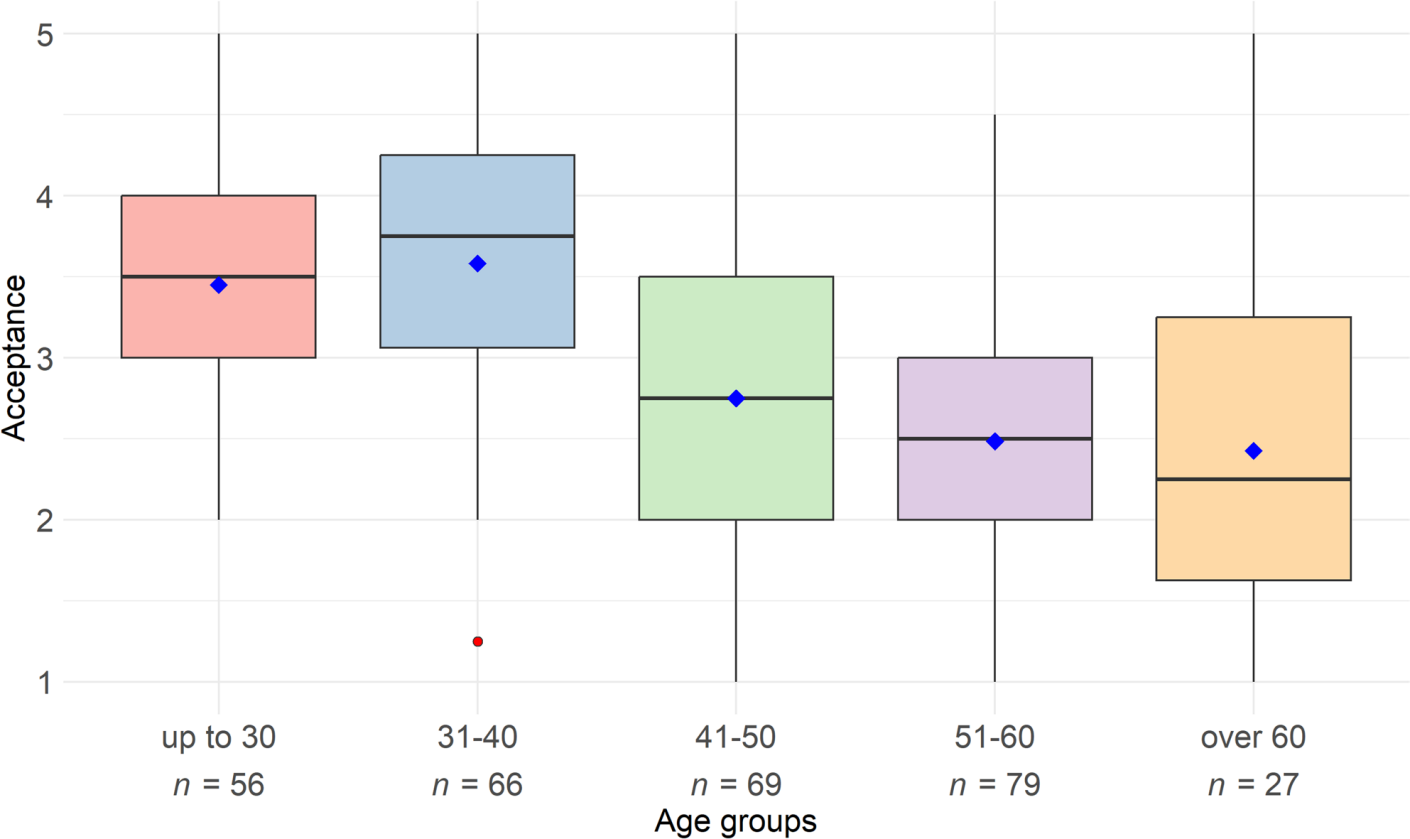
Acceptance of digital health technologies among physiotherapists by age groups (*N* = 297). Note: The boxplots show the distribution of acceptance values for different age groups. Acceptance was measured on a five-point Likert scale, with higher values indicating stronger acceptance. Mean values are represented by blue diamonds, and outliers by red dots

The analysis of acceptance across age groups revealed significant differences among the various age categories. The group aged 31–40 demonstrated the highest level of acceptance, followed by those up to 30 years (see Fig. 1). The acceptance of DHT progressively decreased with age, reaching its lowest point in the group older than 60 years.

A Shapiro-Wilk test was conducted to assess the normality of the acceptance scores within the age groups. The results indicated that the assumption of normality was violated in the 31-40-year-old group (*p* = .031), while no significant deviations were observed in the other groups. Levene’s test for homogeneity of variance showed no significant violations (*p* = .093), confirming that the assumptions for an ANOVA were met.

The analysis revealed a significant difference in acceptance across the age groups (*F*(4, 292) = 23.4, *p* < .001). Post-hoc analyses using the Tukey-HSD test showed that acceptance in the older age groups (41–50 years, 51–60 years, and older than 60 years) was significantly lower than in the group up to 30 years (*p* < .001). Compared to the 31-40-year-old group, the acceptance levels were also significantly lower in the 41–50 years, 51–60 years, and older than the 60 years groups (*p* < .001). No significant differences were observed between the older age groups (41–50 years, 51–60 years, and older than 60 years). Pearson correlation analyses confirmed the findings, showing a significant negative correlation of *r* = -.45 (*t*(295) = -8.75, *p* < .001, 95% CI [-0.54; -0.36]) between the variables acceptance and age.

### Acceptance of digital tools based on age of physiotherapists

The analysis of the usage of various digital tools, including online courses, sensors, smartphone apps, video consultations, VR, wearables, and web apps revealed age-dependent differences in the frequency of use of certain tools. Due to the low number of responses in some categories, Fisher’s Exact Test was used as it provides robust results even for small sample sizes. Fisher’s Exact Test indicated significant differences in the use of sensors (*p* = .002), wearables (*p* = .002), and web apps (*p* = .008) across the age groups. Additionally, a significant age difference was found for the non-use of digital tools (*p* < .001). No significant differences were found for the use of online courses (*p* = .676), smartphone apps (*p* = .097), VR (*p* = .063), or video consultations (*p* = .519) across the age groups.

**Fig. 2:**
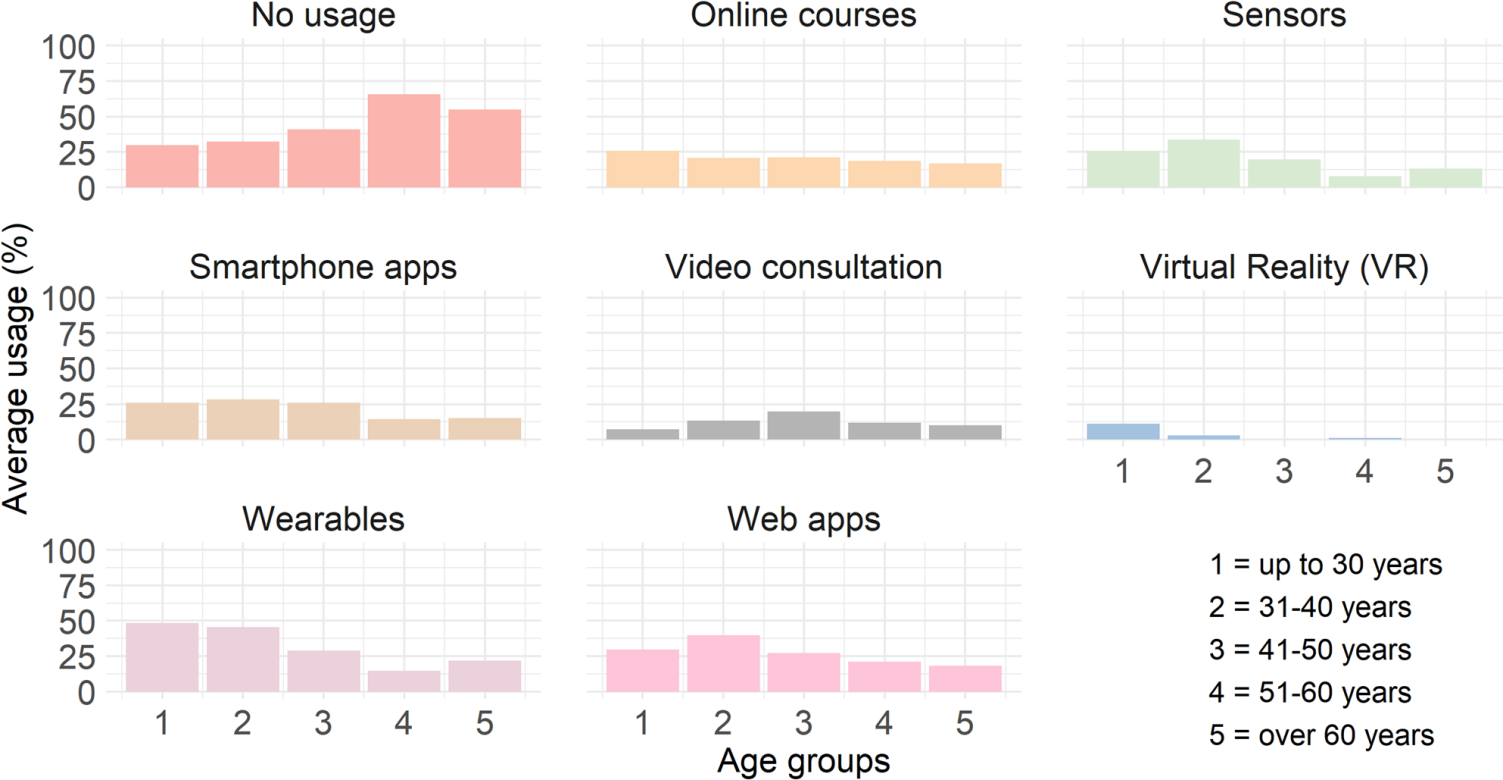
Usage of digital health technologies among physiotherapists by age groups (*N* = 297). Note: The histograms show the average self-reported prior use of digital health technologies in percentage of various digital health tools by age group in the daily practice of physiotherapists

To identify the specific age group differences, pairwise Fisher’s Exact Tests were conducted. The results indicated that non-use of digital tools varied significantly between the up to 30 and 51–60 years (*p* = .005) and 31–40 years and 51–60 years age groups (*p* < .001). For web apps, significantly lower use was observed in the 31-40-year-old group compared to the 51-60-year-old group (*p* < .001). For wearables, significant differences were found between the age groups up to 30 and 41–50 years (*p* = .014), up to 30 and 51–60 years (*p* < .001), and 31–40 years and 51–60 years (*p* = .008). Significant differences in sensor use were shown between the age groups up to 30 and 51–60 years (*p* = .005), 31–40 years and 51–60 years (*p* < .001), and 41–50 years and 51–60 years (*p* = .024). These results suggest that the use of sensors decreases with increasing age. Figure 2 illustrates the mean use of digital tools by age group.

### The impact of gender on the acceptance of digital health technologies

**Fig. 3:**
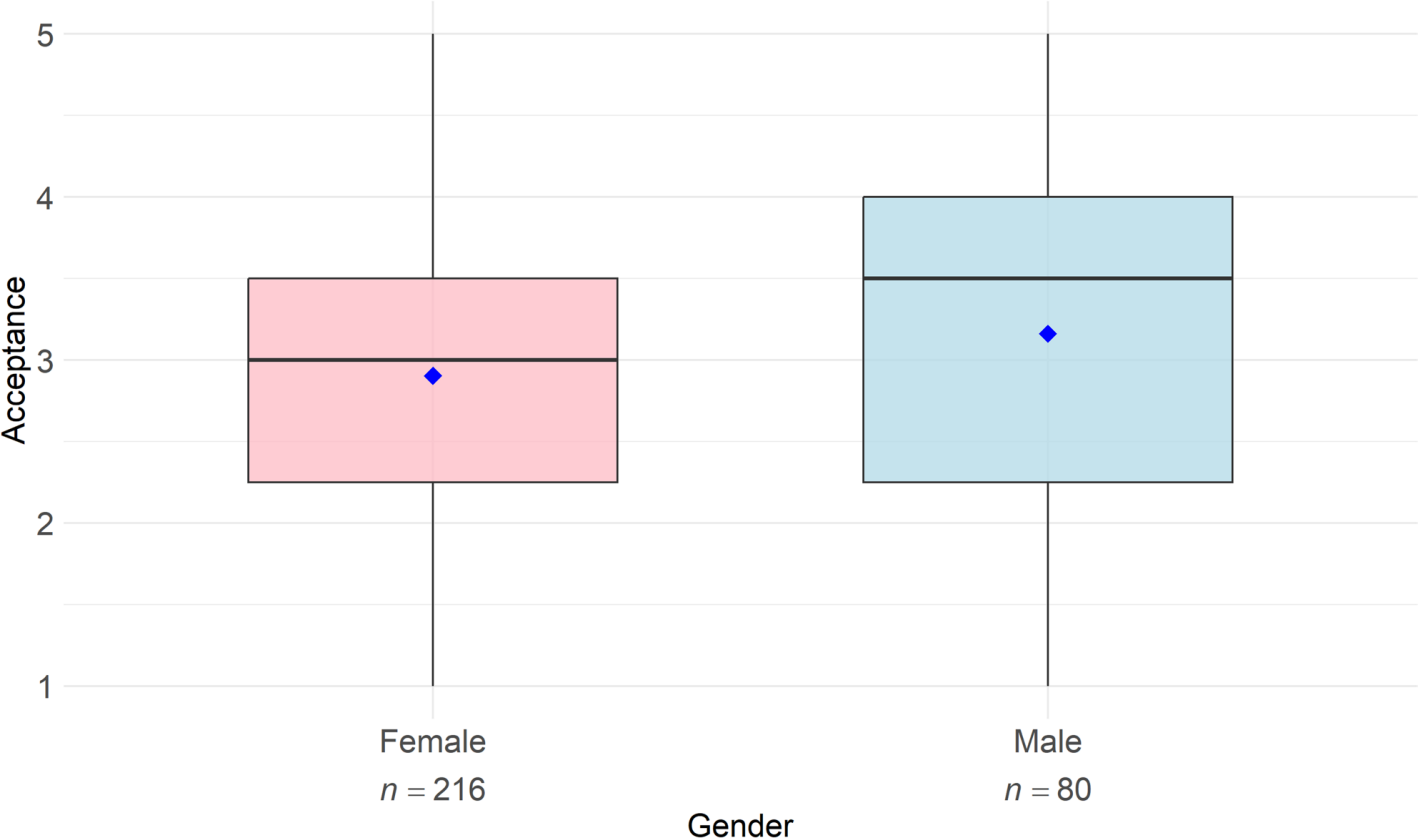
Acceptance of digital health technologies among physiotherapists by gender (*n* = 296). Note: The boxplots illustrate the median and dispersion of acceptance values by gender among physiotherapists. Mean values are indicated by blue diamonds. Acceptance was measured on a five-point Likert scale ranging from 1 (low acceptance) to 5 (high acceptance). One participant did not specify a gender and was excluded from the gender-based analysis

An analysis of gender and its impact on the acceptance of DHT was conducted. One participant was excluded from the analysis due to missing gender specification. Descriptive analyses revealed that female physiotherapists (*n* = 216) exhibited a lower mean score for acceptance of digital technologies (*M* = 2.90, *SD* = 0.91) compared to their male counterparts (*n* = 80; *M* = 3.16, *SD* = 1.09), as depicted in Fig. 3. A subsequent Mann-Whitney U test confirmed significant mean differences between female and male physiotherapists (*U* = 7122, *p* = .020).

### Impact of professional technology use on the acceptance of digital health technologies

**Fig. 4:**
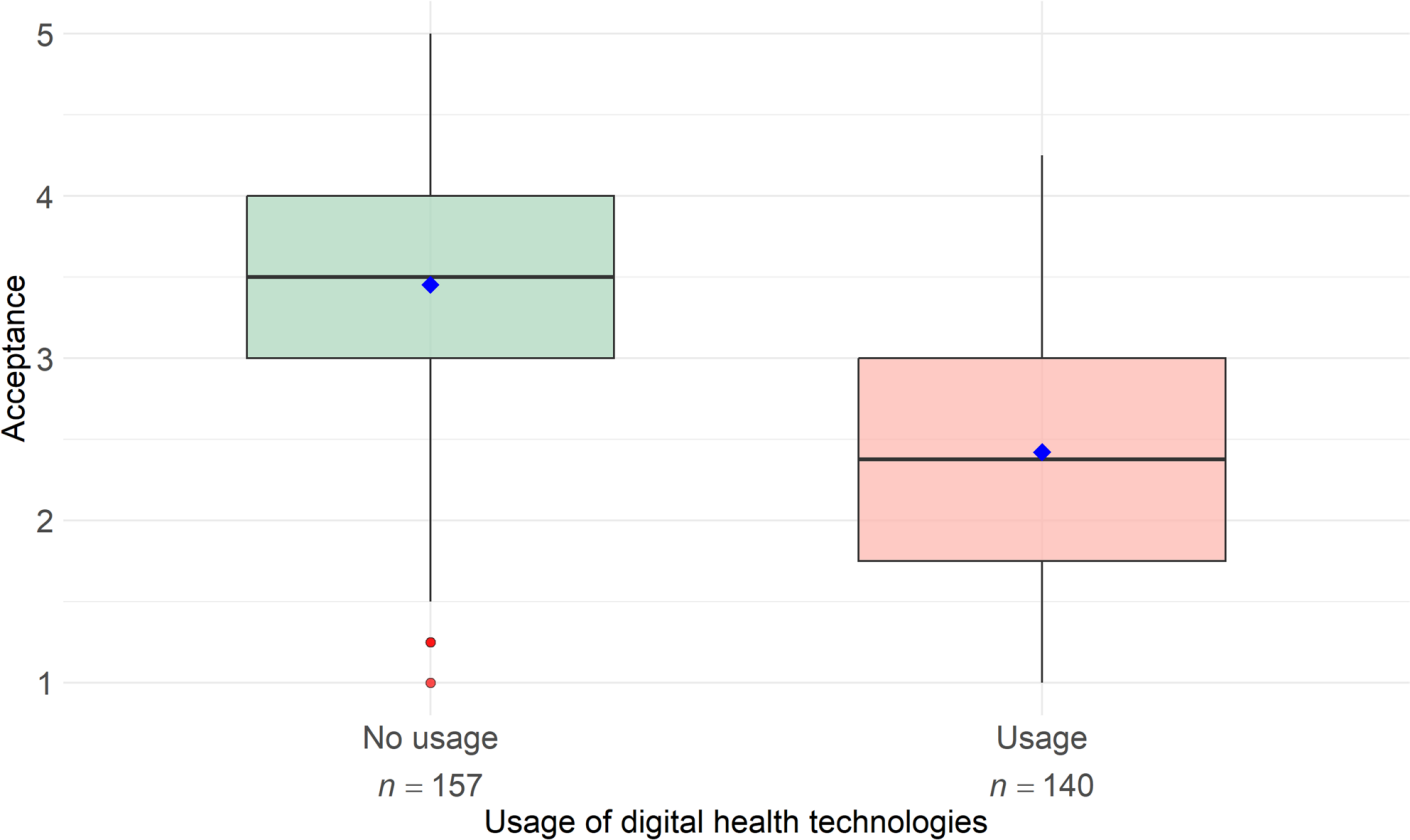
Acceptance of digital health technologies among physiotherapists based on use experience in practice (*N* = 297). Note: The boxplots illustrate the median and dispersion of acceptance values (intention to use, mean scores) based on a five-point Likert scale ranging from 1 (low acceptance) to 5 (high acceptance). Results are shown separately for physiotherapists who reported prior professional use of digital health technologies and those who did not. Mean values are represented by blue diamonds, while outliers are indicated by red dots

The analysis of acceptance scores between physiotherapists who reported using technology in their work (*n* = 157) and those who did not (*n* = 140) revealed that the non-users had higher acceptance of DHT (*M* = 3.45, *SD* = 0.83) compared to the users (*M* = 2.42, *SD* = 0.82). Given the violation of the normality assumption (Shapiro-Wilk test: *p* < .001), the Mann-Whitney U test was employed to examine the difference in acceptance between the two groups, which revealed a significant difference (*U* = 17793.00; *p* < .001) in acceptance between the groups, suggesting that physiotherapists who use DHT in their practice exhibit a substantially lower level of acceptance compared to those who do not employ such technologies (Fig. 4).

### Acceptance based on physiotherapy work settings

**Fig. 5:**
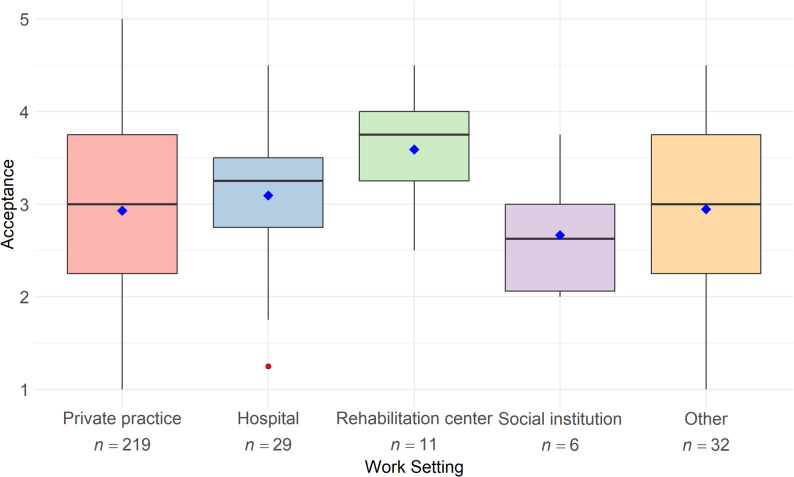
Acceptance of digital health technologies among physiotherapists in different work settings (*N* = 297). Note: The boxplots illustrate the median and dispersion of acceptance scores across different work settings of physiotherapists. Acceptance was measured on a five-point Likert scale ranging from 1 (strongly disagree) to 5 (strongly agree). Mean values are represented by blue diamonds, while outliers are indicated by red dots

The ANOVA examined acceptance across different work settings revealed no significant difference in acceptance between the settings of hospital, private practice, rehabilitation facility, social institution, and others (*F*(4, 292) = 1.497, *p* = .203). The acceptance scores are generally similar across the different settings (see Fig. 5).

## Discussion

The aim of this UTAUT-based survey study was to identify determinants of physiotherapists’ acceptance of DHT and to examine whether age, gender, actual use, and work setting are associated with acceptance levels.

Acceptance of DHT was positively associated with the UTAUT determinants PE, SI, and FC, as well as with formal education in digital health. In contrast, age and DHT use showed significant negative associations with DHT acceptance, whereas EE, knowledge, technology readiness, and eHealth literacy did not contribute to the explained variance. While a negative correlation with age and the limited explanatory value of additional UTAUT-related determinants align with previous research, the finding that self-reported DHT experience was associated with lower acceptance was unexpected and is examined in detail below (see section “*Actual professional use of digital health technologies*”).

Overall, these findings highlight the relevance of key UTAUT constructs such as PE, SI, and FC for understanding the acceptance of DHT, as has already been demonstrated among medical professionals [59–62] and, to the best of our knowledge, for physiotherapists for the first time. In line with the general UTAUT literature and specifically in the eHealth domain [53], PE was the strongest determinant of acceptance in our study. Regarding PE, studies have shown that healthcare professionals develop a greater motivation to use digital technologies when they are convinced that these offer practical benefits and make their work easier [59]. A stronger perception of practical benefits can thus promote acceptance. Similarly, studies on FC have found that the implementation of DHT into practical activities and the provision of technical and organizational resources by medical institutions may facilitate acceptance of DHT in the medical workplace [61]. Regarding SI, the role of employers and colleagues, whose support can increase the acceptance of DHT, was especially highlighted [63]. The moderator of formal education in digital health, which complements the UTAUT constructs, also showed a significant positive impact on acceptance. A greater integration of digital technologies into medical curricula and training programs may contribute to higher acceptance [64].

### Negative influencing factors on the acceptance of digital health technologies

#### Age of physiotherapists

The inverse relationship between age and technology acceptance confirms earlier findings across different age groups [65], although the specific target group of physiotherapists has been less studied. The analysis of age groups shows that the acceptance of DHT significantly decreases with age. The 31-to 40-year-olds showed the highest acceptance, followed by the youngest age group (up to 30 years). These results may reflect a higher affinity for technology and greater familiarity with digital tools in the younger groups, as these age groups can be considered as Digital Natives [66, 67]. Digital Natives, defined as individuals born after 1980 who have grown up with digital technologies, are characterized by their early and natural familiarity with digital tools [67]. Consequently, younger physiotherapists who align with this profile may exhibit a reduced degree of hesitancy in leveraging digital technologies within medical settings. Considering the predictive factor of SI, colleagues (particularly Digital Natives) may serve as a resource for older physiotherapists and could support DHT use, which may in turn be linked to higher acceptance. In contrast, with increasing age and professional experience, physiotherapists may be more likely to transition into administrative, managerial, or leadership roles rather than continuing direct patient care. Consequently, they might have less exposure to emerging approaches such as eHealth. Additionally, Zhou and colleagues [65] found that older age groups expressed more concerns about data privacy, rated the current use of digital technologies in healthcare as insufficient and showed little interest in their implementation.

***Use of digital tools: Differences across age groups.*** As mentioned earlier, DHT includes a variety of different tools, such as digital organizational applications, telemedicine, and smartphone apps. A differentiated view of the tools across age groups provides valuable insights into which technologies are used by physiotherapists and which tools show lower usage rates. The results show that a large proportion of physiotherapists reported not using DHT for professional purposes, with non-use being significantly higher in older age groups. When DHT are used, differences in usage between the age groups become apparent. Younger physiotherapists use tools like sensors, wearables, and web apps more frequently in their professional practice than older physiotherapists. In contrast, no significant differences across age groups were found in the usage of smartphone apps, online courses, and telemedicine. The use of VR is rare across all age groups. In summary, the results suggest that some DHT are not yet widespread among physiotherapists, and acceptance of these technologies varies across age groups.

In an earlier study, only 36% of healthcare professionals in Germany, including physiotherapists, speech therapists, and occupational therapists, reported knowing about DiGA [40]. Insufficient knowledge and low experience have been identified in earlier research as possible causes for low digital use in healthcare professions [68, 69]. A recent review by Sia et al. [69] highlights that physiotherapists lack appropriate training to handle DHT. Our results confirm the positive impact of formal education in digital health and offer important implications for designing curricula in physiotherapy education and future training programs. On the one hand, content on DHT could be integrated into physiotherapy education. On the other hand, practicing physiotherapists could receive targeted training on the use and application of DHT through preferred channels. As older professionals seem to be more receptive to online courses and telemedicine, online training could be an effective way to convey knowledge about DHT. At the same time, further research is needed to identify the formats that best support physiotherapists in using and accepting digital tools. Such studies could help develop tailored training programs that meet the specific needs of different age groups and thus promote the widespread integration of DHT into physiotherapy practice.

#### Actual professional use of digital health technologies

An unexpected finding of our study was that acceptance values were significantly higher among non-users compared to physiotherapists reporting prior professional use of DHT. The comparatively lower acceptance among physiotherapists with DHT use experience may partly be explained by a higher proportion of female physiotherapists in our study, who reported lower average acceptance of DHT overall. It is also possible that professionals with use experience have more concrete insights into negative aspects of DHT in daily physiotherapy practice, such as actually experienced technical issues or low acceptance among patients. This pattern is consistent with findings from implementation research, suggesting that individuals with direct experience of innovation often evaluate it more critically based on its practical fit, reliability, and added workload rather than on anticipated benefits alone [70]. From a post-adoption perspective, this finding may also reflect a mismatch between initial expectations and actual experiences with DHT [71]. While non-users may primarily base their attitudes on anticipated benefits or social desirability bias, users evaluate DHT in real-world practice, where technical, organizational, and workflow-related challenges may become apparent. If perceived benefits fail to meet prior expectations, acceptance may decrease after initial use [72]. A recent survey of Norwegian physiotherapists identified insufficient technical infrastructure, such as inadequate internet connection or malfunctioning devices and software, as a key barrier to DHT use [73]. Qualitative results and systematic reviews further report limited resources and perceived poor outcomes as hindering factors for physiotherapists [66, 74]. In addition, available DHT may not meet physiotherapists’ expectations regarding usability or therapeutic benefit [75]. Such discrepancies between expectations and real-world performance have been described as a common source of disillusionment following the adoption of digital health innovations [70]. These barriers predominantly affect actual users and may, through repeated exposure, lead to frustration and reduced acceptance. Future studies should therefore focus on perceived barriers among physiotherapists with direct experience of DHT to better understand context-specific hindering factors within the German healthcare system.

### Other influencing factors on acceptance of digital health technologies

In contrast to other studies [45, 68], we found a significant difference in DHT acceptance between female and male physiotherapists, with male physiotherapists reporting higher acceptance levels. Descriptive analyses of DHT use and application areas further showed that men also integrated DHT into their practice more frequently. As potential explanations for the observed gender differences, previous research on digital health suggests that men may exhibit a greater affinity for technology, which in some professional settings has been associated with higher acceptance and use of eHealth and telemedicine applications [76].

While previous research has shown that the work setting [73] impacts the acceptance of DHT among physiotherapists, we found no effects of the factors investigated on acceptance. However, a selective set of variables beyond the UTAUT framework was included to limit participant burden. This approach was chosen due to anticipated recruitment challenges in healthcare professionals and the limited evidence base on technology acceptance among physiotherapists. Future studies could incorporate other potentially relevant constructs from other contexts of acceptance research, such as the extended UTAUT (UTAUT2; [77]) or the Technology Acceptance Model (TAM; [78]).

### Implications for practice

The findings suggest that training and technical support may need to be tailored to the needs of different age groups. For older physiotherapists, low-threshold entry offers, and intensive support may be helpful, while younger physiotherapists could be more motivated by the provision of concrete application opportunities. Furthermore, strategies should be developed to address unrealistic expectations and avoid overwhelming professionals. Ultimately, it is necessary to identify perceived barriers to the use of DHT among German healthcare professionals to develop strategies to address these barriers and support the use and acceptance of DHT. Given that PE was the strongest determinant, information and education could focus on the benefits of digital health technologies, emphasizing their usefulness even when there is no extra compensation for using DHT, and may help foster intrinsic motivation.

### Strengths, limitations, and future research

The sample predominantly consists of female participants, which reflects the gender distribution in healthcare professions in Germany [8]. This enhances the representativeness of our findings for the target population. However, future studies with a more gender-balanced sample could provide further insights into potential differences in the use and acceptance of DHT among male professionals. The high variance explained by our model shows the strength of the predictors considered. The average age of the sample was 45 years (*M* = 44.6, *SD* = 12.3, range = 21–76 years), which is very close to the national average for the general population of German physiotherapists [79]. Furthermore, the majority of participants worked in outpatient practices, followed by hospitals, which closely reflects the national distribution reported by the German Federal Statistical Office [8]. This further supports the representativeness of our sample and strengthens the external validity of our findings. Another strength of this study lies in the theory-driven approach using an extended UTAUT model, which explained a substantial proportion of variance in DHT acceptance. The high explanatory power underscores the relevance of the selected constructs for understanding technology acceptance processes among physiotherapists.

Despite these strengths, several limitations should be acknowledged. It is important to note that data collection was based on a self-report instrument, meaning responses were derived from participants’ subjective assessments, such as their use of digital tools. Self-reports are susceptible to biases, including social desirability and potential recall issues. Incorporating objective measures, such as the technical infrastructure of physiotherapy practices, actual use statistics of DHT, or qualitative data on usage barriers and work context, could provide a more comprehensive and accurate assessment of actual use and acceptance. Beyond individual acceptance, such as reimbursement models, regulatory policies, and interoperability challenges, which may significantly influence the widespread implementation of DHT in physiotherapy. Another limitation of this study is that specific reasons underlying the use or non-use of digital health technologies were not assessed, as identifying barriers to DHT use was not the primary focus of the survey. Consequently, interpretations regarding the lower acceptance among DHT users remain exploratory and should be examined in more detail in future research. Longitudinal studies may help to examine how acceptance of digital health solutions evolves over time and how sustained exposure, training, and institutional support may enhance acceptance and digital readiness. Furthermore, expanding investigations to include diverse healthcare settings and international comparisons could provide a more comprehensive understanding of the structural and cultural determinants of DHT acceptance. Additionally, the rapid advancement of AI-driven applications and telehealth solutions warrants further exploration regarding their impact on clinical workflows, patient outcomes, and professional-patient interactions. By addressing these aspects, future research may contribute to the development of a more holistic framework for the successful and sustainable integration of digital health technologies in physiotherapy and the broader healthcare landscape.

### Conclusion and outlook

This study underscores the multifaceted factors influencing the acceptance of DHT among physiotherapists. Our findings highlight the role of PE, SI, FC, and formal education in digital health in shaping technology acceptance. These insights contribute to the growing body of research on digital transformation in healthcare and highlight the potential value of targeted strategies to support the integration of DHT into physiotherapy practice.

## Data Availability

The datasets generated and analyzed during the current study are available from the corresponding author upon reasonable request.

## Abbreviations

AI: Artificial Intelligence
BfArM: Federal Institute for Drugs and Medical Devices (German “Bundesinstitut für Arzneimittel und Medizinprodukte“)
DiGA: Digital Health Applications (German “Digitale Gesundheitsanwendungen“)
DHT: Digital Health Technologies
DTx: Digital Therapeutics
EE: Effort expectancy
EHR: Electronic health records
FC: Facilitating conditions
G-eHEALS: German adaptation of the eHealth Literacy Scale
MSD: Musculoskeletal disorders
PE: Performance expectancy
SI: Social influence
UTAUT: Unified Theory of Acceptance and Use of Technology
VR: Virtual Reality

## References

[1] Narenthiran P, Granville Smith I, Williams FMK. Does the addition of manual therapy to exercise therapy improve pain and disability outcomes in chronic low back pain: a systematic review. J Bodyw Mov Ther. 2025 June;42:146–52. 10.1016/j.jbmt.2024.12.004 40325660

[2] Grenier JP, Rothmund M. A critical review of the role of manual therapy in the treatment of individuals with low back pain. J Man Manipulative Therapy. 2024 Sept;32(2):464–77. 10.1080/10669817.2024.2316393 PMC11421166 38381584

[3] World Health Organization. World Health Organization. 2022 [cited 2025 Feb 27]. Musculoscletal Health. Available from: https://www.who.int/news-room/fact-sheets/detail/musculoskeletal-conditions.

[4] Ferreira ML, De Luca K, Haile LM, Steinmetz JD, Culbreth GT, Cross M, et al. Global, regional, and National burden of low back pain, 1990–2020, its attributable risk factors, and projections to 2050: a systematic analysis of the global burden of disease study 2021. Lancet Rheumatol. 2023 June;5(6):e316–29. 10.1016/S2665-9913(23)00098-X PMC10234592 37273833

[5] Waltersbacher A. Heilmittelbericht 2022/2023 [Internet]. Wissenschaftliches Institut der AOK (WldO); 2023 Jan [cited 2025 Jan 24]. Available from: https://www.wido.de/fileadmin/Dateien/Dokumente/Publikationen_Produkte/Buchreihen/Heilmittelbericht/wido_hei_heilmittelbericht_2022_2023.pdf#:~:text=Die%20h%C3%A4ufigste%20Diagnose%20f%C3%BCr%20eine%20sprachtherapeutische%20Behandlung%20war%20mit%20einem. Abgerufen am 24.01.2025.

[6] Kopkow C, Garsch S. Physiotherapeutische Versorgungssituation in Deutschland: Analyse der Leistungen aus dem Heilmittelkatalog der physikalischen Therapie von 2007 bis 2016 und Prognose für 2026. In: Deutsches Netzwerk Versorgungsforschung e V [Internet]. Berlin: German Medical Science; 2018 [cited 2025 Jan 25]. Available from: https://www.egms.de/static/de/meetings/dkvf2018/18dkvf004.shtml.

[7] Hillienhof A, Gesundheitsberufe. Fachkräftemangel größer als angenommen. Dtsch Arztebl International [Internet]. 2018 Sept 4 [cited 2025 Jan 20]; Available from: https://www.aerzteblatt.de/archiv/201695/Gesundheitsberufe-Fachkraeftemangel-groesser-als-angenommen.

[8] Bundesamt S. Destatis. 2025 [cited 2025 Mar 18]. Gesundheitspersonal: Deutschland, Jahre, Einrichtungen, Geschlecht, Berufe im Gesundheitswesen. Available from: https://www-genesis.destatis.de/datenbank/online/statistic/23621/table/23621-0002/search/s/cGh5c2lvdGhlcmFwaWU=.

[9] El Aoufy K, Melis MR, Magi CE, Bellando-Randone S, Tamburini M, Bandini G, et al. Evidence for telemedicine heterogeneity in rheumatic and musculoskeletal diseases care: a scoping review. Clin Rheumatol. 2024 Sept;43(9):2721–63. 10.1007/s10067-024-07052-w PMC11330403 38985235

[10] León-Salas B, González‐Hernández Y, Infante‐Ventura D, De Armas‐Castellano A, García‐García J, García‐Hernández M, et al. Telemedicine for neurological diseases: a systematic review and meta‐analysis. Euro J Neurol. 2023;30(1):241–54. 10.1111/ene.15599 36256522

[11] Marziniak M, Brichetto G, Feys P, Meyding-Lamadé U, Vernon K, Meuth SG. The use of digital and remote communication technologies as a tool for multiple sclerosis management: narrative review. JMIR Rehabil Assist Technol. 2018;5(1):e5. 29691208 10.2196/rehab.7805 PMC5941090

[12] Sakima A, Akagi Y, Akasaki Y, Fujii T, Haze T, Kawakami-Mori F, et al. Effectiveness of digital health interventions for telemedicine/telehealth for managing blood pressure in adults: a systematic review and meta-analysis. Hypertens Res. 2025;48(2):478–91. 38977877 10.1038/s41440-024-01792-7

[13] Hayashi K, Miki K, Shiro Y, Tetsunaga T, Takasusuki T, Hosoi M, et al. Utilization of telemedicine in conjunction with wearable devices for patients with chronic musculoskeletal pain: a randomized controlled clinical trial. Sci Rep. 2025;15(1):1396. 39789122 10.1038/s41598-024-85056-x PMC11718130

[14] Park Y, Kim EJ, Park S, Lee M. Digital health intervention effect on older adults with chronic diseases living alone: systematic review and Meta-Analysis of randomized controlled trials. J Med Internet Res. 2025;27:e63168. 40163849 10.2196/63168 PMC11997531

[15] Carpenter AB, Sheppard E, Atabaki S, Shur N, Tigranyan A, Benchoff T, et al. A symposium on the clinic of the future and telehealth: highlights and future directions. Cureus [Internet]. 2021 May 25 [cited 2025 Mar 11]; Available from: https://www.cureus.com/articles/57333-a-symposium-on-the-clinic-of-the-future-and-telehealth-highlights-and-future-directions. 10.7759/cureus.15234 PMC8223952 34178544

[16] Garcia JP, Avila FR, Torres-Guzman RA, Maita KC, Lunde JJ, Coffey JD, et al. A narrative review of telemedicine and its adoption across specialties. mHealth. 2024;10:19–19. 38689613 10.21037/mhealth-23-28 PMC11058596

[17] Ferorelli D, Moretti L, Benevento M, Mastrapasqua M, Telegrafo M, Solarino B, et al. Digital health Care, Telemedicine, and medicolegal issues in orthopedics: A review. IJERPH. 2022;19(23):15653. 36497728 10.3390/ijerph192315653 PMC9735483

[18] Youssef Y, De Wet D, Back DA, Scherer J. Digitalization in orthopaedics: a narrative review. Front Surg. 2024;10:1325423. 38274350 10.3389/fsurg.2023.1325423 PMC10808497

[19] Ackerley S, Wilson N, Boland P, Read J, Connell L. Implementation of neurological group-based telerehabilitation within existing healthcare during the COVID-19 pandemic: a mixed methods evaluation. BMC Health Serv Res. 2023 June 21;23(1):671. 10.1186/s12913-023-09635-w PMC10283243 37344774

[20] Zhou T, Zhang S, Liu S, Yu J. Digital technology integration in home-based exercise: a systematic review of research evolution, applications, and impact mechanisms. BMC Public Health. 2025;25(1):3528. 41107883 10.1186/s12889-025-24679-9 PMC12534984

[21] Cottrell MA, Russell TG. Telehealth for musculoskeletal physiotherapy. Musculoskelet Sci Pract. 2020;48:102193. 32560876 10.1016/j.msksp.2020.102193 PMC7261082

[22] Merolli M, Francis JJ, Vallance P, Bennell KL, Malliaras P, Hinman RS. Evaluation of Patient-Facing mobile apps to support physiotherapy care: systematic review. JMIR Mhealth Uhealth. 2024;12:e55003. 38437018 10.2196/55003 PMC10949126

[23] Sandal LF, Bach K, Øverås CK, Svendsen MJ, Dalager T, Stejnicher Drongstrup Jensen J, et al. Effectiveness of App-Delivered, tailored Self-management support for adults with lower back Pain–Related disability: A self BACK randomized clinical trial. JAMA Intern Med. 2021;181(10):1288. 34338710 10.1001/jamainternmed.2021.4097 PMC8329791

[24] Richardson BR, Truter P, Blumke R, Russell TG. Physiotherapy assessment and diagnosis of musculoskeletal disorders of the knee via telerehabilitation. J Telemed Telecare. 2017;23(1):88–95. 26985005 10.1177/1357633X15627237

[25] Lamplot JD, Pinnamaneni S, Swensen-Buza S, Lawton CD, Dines JS, Nawabi DH, et al. The virtual shoulder and knee physical examination. Orthop J Sports Med. 2020;8(10):2325967120962869. 33614791 10.1177/2325967120962869 PMC7871077

[26] Grona SL, Bath B, Busch A, Rotter T, Trask C, Harrison E. Use of videoconferencing for physical therapy in people with musculoskeletal conditions: a systematic review. J Telemed Telecare. 2018 June;24(5):341–55. 10.1177/1357633X17700781 28403669

[27] Levy CE, Silverman E, Jia H, Geiss M, Omura D. Effects of physical therapy delivery via home video telerehabilitation on functional and health-related quality of life outcomes. J Rehabil Res Dev. 2015;52(3):361–70. 26230650 10.1682/JRRD.2014.10.0239

[28] Kloek CJJ, Van Dongen JM, De Bakker DH, Bossen D, Dekker J, Veenhof C. Cost-effectiveness of a blended physiotherapy intervention compared to usual physiotherapy in patients with hip and/or knee osteoarthritis: a cluster randomized controlled trial. BMC Public Health. 2018;18(1):1082. 30170586 10.1186/s12889-018-5975-7 PMC6119267

[29] Blixt L, Solbrække KN, Bjorbækmo WS. Physiotherapists’ experiences of adopting an eTool in clinical practice: a post-phenomenological investigation. Physiotherapy Theory Pract 2021 Sept 2;37(9):1005–17. 10.1080/09593985.2019.1681042 31635512

[30] Danbjørg DB, Villadsen A, Gill E, Rothmann MJ, Clemensen J. Usage of an exercise app in the care for people with osteoarthritis: User-Driven exploratory study. JMIR Mhealth Uhealth. 2018;6(1):e11. 29326092 10.2196/mhealth.7734 PMC5785680

[31] Button K, Nicholas K, Busse M, Collins M, Spasić I. Integrating self-management support for knee injuries into routine clinical practice: TRAK intervention design and delivery. Musculoskelet Sci Pract. 2018;33:53–60. 29172113 10.1016/j.msksp.2017.11.002

[32] Hinman RS, Nelligan RK, Bennell KL, Delany C. Sounds a bit Crazy, but it was almost more personal: A qualitative study of patient and clinician experiences of physical Therapist–Prescribed exercise for knee osteoarthritis via skype. Arthritis Care Res. 2017;69(12):1834–44. 10.1002/acr.23218 28217864

[33] Lambert TE, Harvey LA, Avdalis C, Chen LW, Jeyalingam S, Pratt CA, et al. An app with remote support achieves better adherence to home exercise programs than paper handouts in people with musculoskeletal conditions: a randomised trial. J Physiotherapy. 2017 July;63(3):161–7. 10.1016/j.jphys.2017.05.015 28662834

[34] Revenäs Å, Opava CH, Martin C, Demmelmaier I, Keller C, Åsenlöf P. Development of a Web-Based and mobile app to support physical activity in individuals with rheumatoid arthritis: results from the second step of a Co-Design process. JMIR Res Protoc. 2015;4(1):e22. 25665589 10.2196/resprot.3795 PMC4342685

[35] Bennell KL, Nelligan R, Dobson F, Rini C, Keefe F, Kasza J, et al. Effectiveness of an Internet-Delivered exercise and Pain-Coping skills training intervention for persons with chronic knee pain: A randomized trial. Ann Intern Med. 2017;166(7):453–62. 28241215 10.7326/M16-1714

[36] Huber S, Priebe JA, Baumann KM, Plidschun A, Schiessl C, Tölle TR. Treatment of low back pain with a digital multidisciplinary pain treatment app: Short-Term results. JMIR Rehabil Assist Technol. 2017;4(2):e11. 29203460 10.2196/rehab.9032 PMC5735251

[37] Lawford BJ, Delany C, Bennell KL, Hinman RS. I was really sceptical… But it worked really well: a qualitative study of patient perceptions of telephone-delivered exercise therapy by physiotherapists for people with knee osteoarthritis. Osteoarthritis and Cartilage. 2018 June;26(6):741–50. 10.1016/j.joca.2018.02.909 29572130

[38] Corbetta D, Imeri F, Gatti R. Rehabilitation that incorporates virtual reality is more effective than standard rehabilitation for improving walking speed, balance and mobility after stroke: a systematic review. J Physiotherapy. 2015 July;61(3):117–24. 10.1016/j.jphys.2015.05.017 26093805

[39] Goeldner M, Gehder S. Digital health applications (DiGAs) on a fast track: insights from a Data-Driven analysis of prescribable digital therapeutics in Germany from 2020 to Mid-2024. J Med Internet Res. 2024;26:e59013. 39208415 10.2196/59013 PMC11393499

[40] Frey S, Kerkemeyer L. Acceptance of digital health applications in non-pharmacological therapies in German statutory healthcare system: results of an online survey. Digit HEALTH. 2022;8:205520762211311. 10.1177/20552076221131142 PMC9732791 36506488

[41] Fassbender A, Donde S, Silva M, Friganovic A, Stievano A, Costa E, et al. Adoption of digital therapeutics in Europe. TCRM. 2024;20:939–54. 10.2147/TCRM.S489873 PMC11687304 39741688

[42] Bundesinstitut für Arzneimittel und Medizinprodukte. DiGA-Verzeichnis. 2025. DiGA-Verzeichnis. Available from: https://diga.bfarm.de/de/verzeichnis.

[43] Agnew JMR, Hanratty CE, McVeigh JG, Nugent C, Kerr DP. An investigation into the use of mHealth in musculoskeletal physiotherapy: scoping review. JMIR Rehabil Assist Technol. 2022;9(1):e33609. 35275089 10.2196/33609 PMC8956993

[44] Keel S, Schmid A, Keller F, Schoeb V. Investigating the use of digital health tools in physiotherapy: facilitators and barriers. Physiother Theory Pract. 2023 July;3(7):1449–68. 10.1080/09593985.2022.2042439 35293846

[45] Estel K, Scherer J, Dahl H, Wolber E, Forsat ND, Back DA. Potential of digitalization within physiotherapy: a comparative survey. BMC Health Serv Res. 2022;22(1):496. 35418069 10.1186/s12913-022-07931-5 PMC9007581

[46] Tivian XIGH. Unipark [Internet]. 2023. Available from: https://www.unipark.com.

[47] Venkatesh M. Davis, Davis. User acceptance of information technology: toward a unified view. MIS Q. 2003;27(3):425.

[48] Hennemann S, Beutel ME, Zwerenz R. Ready for eHealth? Health professionals’ acceptance and adoption of eHealth interventions in inpatient routine care. J Health Communication. 2017;22(3):274–84. 28248626 10.1080/10810730.2017.1284286

[49] Liu L, Miguel Cruz A, Rios Rincon A, Buttar V, Ranson Q, Goertzen D. What factors determine therapists’ acceptance of new technologies for rehabilitation – a study using the unified theory of acceptance and use of technology (UTAUT). Disabil Rehabil. 2015;37(5):447–55. 24901351 10.3109/09638288.2014.923529

[50] Soellner R, Huber S, Reder M. The concept of eHealth literacy and its measurement: German translation of the eHEALS. J Media Psychol. 2014;26(1):29–38.

[51] Neyer FJ, Felber J, Gebhardt C. Entwicklung und validierung einer Kurzskala Zur erfassung von technikbereitschaft. Diagnostica. 2012;58(2):87–99.

[52] Dingel J, Kleine AK, Cecil J, Sigl AL, Lermer E, Gaube S. Predictors of health care practitioners’ intention to use AI-Enabled clinical decision support systems: Meta-Analysis based on the unified theory of acceptance and use of technology. J Med Internet Res. 2024;26:e57224. 39102675 10.2196/57224 PMC11333871

[53] Philippi P, Baumeister H, Apolinário-Hagen J, Ebert DD, Hennemann S, Kott L, et al. Acceptance towards digital health interventions – Model validation and further development of the unified theory of acceptance and use of technology. Internet Interventions. 2021;26:100459. 34603973 10.1016/j.invent.2021.100459 PMC8463857

[54] Norman CD, Skinner HA. eHEALS: the eHealth literacy scale. J Med Internet Res. 2006;8(4):e27. 17213046 10.2196/jmir.8.4.e27 PMC1794004

[55] Ebert DD, Berking M, Cuijpers P, Lehr D, Pörtner M, Baumeister H. Increasing the acceptance of internet-based mental health interventions in primary care patients with depressive symptoms. A randomized controlled trial. J Affect Disord. 2015;176:9–17. 25682378 10.1016/j.jad.2015.01.056

[56] R Core Team. R: A language and environment for statistical computing [Internet]. R Foundation for Statistical Computing; Available from: https://www.R-project.org/.

[57] Wickham H, Francois R, Henry L, Kirill M, Vaughan D. dplyr: a grammar of data manipulation [Internet]. 2023. Available from: https://dplyr.tidyverse.org.

[58] Wickham H. Ggplot2: elegant graphics for data analysis. New York: Springer; 2009. p. 212. (Use R!).

[59] Alabdullah JH, Van Lunen BL, Claiborne DM, Daniel SJ, Yen C, Gustin TS. Application of the unified theory of acceptance and use of technology model to predict dental students’ behavioral intention to use teledentistry. J Dent Educ. 2020;84(11):1262–9. 32705688 10.1002/jdd.12304

[60] Kohnke A, Cole ML, Bush R. Incorporating UTAUT predictors for Understanding home care patients’ and clinician’s acceptance of healthcare telemedicine equipment. J Technol Manage Innov. 2014 July;9(2):29–41.

[61] Rouidi M, Elouadi AE, Hamdoune A, Choujtani K, Chati A. TAM-UTAUT and the acceptance of remote healthcare technologies by healthcare professionals: a systematic review. Inf Med Unlocked. 2022;32:101008.

[62] Van Der Vaart R, Atema V, Evers AWM. Guided online self-management interventions in primary care: a survey on use, facilitators, and barriers. BMC Fam Pract. 2016;17(1):27. 26961547 10.1186/s12875-016-0424-0 PMC4785635

[63] Vidal-Alaball J, Acosta-Roja R, Pastor Hernández N, Sanchez Luque U, Morrison D, Narejos Pérez S, et al. Telemedicine in the face of the COVID-19 pandemic. Atención Primaria. 2020 June;52(6):418–22. 10.1016/j.aprim.2020.04.003 PMC7164871 32402477

[64] Sahan F, Guthardt L, Panitz K, Siegel-Kianer A, Eichhof I, Schmitt BD, et al. Enhancing digital health awareness and mHealth competencies in medical education: Proof-of-Concept study and summative process evaluation of a quality improvement project. JMIR Med Educ. 2024 Sept 20;10:e59454. 10.2196/59454 PMC11452754 39303285

[65] Zhou L, Bao J, Watzlaf V, Parmanto B. Barriers to and facilitators of the use of mobile health apps from a security perspective: Mixed-Methods study. JMIR Mhealth Uhealth. 2019;7(4):e11223. 30990458 10.2196/11223 PMC6488955

[66] Van Tilburg ML, Spin I, Pisters MF, Staal JB, Ostelo RW, Van Der Velde M, et al. Barriers and facilitators to the implementation of digital health services for people with musculoskeletal conditions in the primary health care setting: systematic review. J Med Internet Res. 2024;26:e49868. 39190440 10.2196/49868 PMC11387918

[67] Helsper EJ, Eynon R. Digital natives: where is the evidence? Br Educational Res J [Internet]. 2013;36(3):503–20.

[68] Kerst A, Zielasek J, Gaebel W. Smartphone applications for depression: a systematic literature review and a survey of health care professionals’ attitudes towards their use in clinical practice. Eur Arch Psychiatry Clin Neurosci. 2020;270(2):139–52. 30607530 10.1007/s00406-018-0974-3

[69] Sia LL, Sharma S, Ing JBM, Kumar S, Singh DKA. Physiotherapists’ perceptions, readiness, enablers, and barriers to use telerehabilitation: A scoping review. BMR. 2024;37(6):1441–54. 10.3233/BMR-240009 PMC11613087 38905032

[70] Greenhalgh T, Robert G, Macfarlane F, Bate P, Kyriakidou O. Diffusion of innovations in service organizations: systematic review and recommendations. Milbank Q. 2004;82(4):581–629. 15595944 10.1111/j.0887-378X.2004.00325.x PMC2690184

[71] Bhattacherjee A. Understanding information systems continuance: an Expectation-Confirmation Model1. MIS quarterly. Sept. 2001;25(1):351–70.

[72] Greenhalgh T, Wherton J, Papoutsi C, Lynch J, Hughes G, A’Court C, et al. Beyond adoption: A new framework for theorizing and evaluating Nonadoption, Abandonment, and challenges to the Scale-Up, Spread, and sustainability of health and care technologies. J Med Internet Res. 2017;19(11):e367. 29092808 10.2196/jmir.8775 PMC5688245

[73] Martinsen L, Østerås N, Moseng T, Tveter AT, Usage. Attitudes, Facilitators, and barriers toward digital health technologies in musculoskeletal care: survey among primary care physiotherapists in Norway. JMIR Rehabil Assist Technol. 2024 Sept 16;11:e54116. 10.2196/54116 PMC11443180 39283661

[74] Kelly M, Fullen BM, Martin D, Bradley C, McVeigh JG. eHealth interventions to support self-management: perceptions and experiences of people with musculoskeletal disorders and physiotherapists - ‘eHealth: it’s TIME’: A qualitative study. Physiother Theory Pract. 2024;40(5):1011–21. 36426843 10.1080/09593985.2022.2151334

[75] Blumenthal J, Wilkinson A, Chignell M. Physiotherapists’ and physiotherapy students’ perspectives on the use of mobile or wearable technology in their practice. Physiotherapy Can. 2018;70(3):251–61. 10.3138/ptc.2016-100.e PMC6158559 30275650

[76] Haluza D, Wernhart A. Does gender matter? Exploring perceptions regarding health technologies among employees and students at a medical university. Int J Med Informatics. 2019;130:103948. 10.1016/j.ijmedinf.2019.08.008 31442846

[77] Venkatesh, Thong. Xu. Consumer acceptance and use of information technology: extending the unified theory of acceptance and use of technology. MIS Q. 2012;36(1):157.

[78] Davis FD, Perceived, Usefulness. Perceived ease of Use, and user acceptance of information technology. MIS Q. 1989 Sept;13(3):319.

[79] Statistisches Bundesamt. Destatis. 2025 [cited 2025 Mar 18]. Gesundheitspersonal (Vollzeitäquivalente): Deutschland, Jahre, Altersgruppen, Berufe im Gesundheitswesen. Available from: https://www-genesis.destatis.de/datenbank/online/statistic/23621/table/23621-0008/search/s/cGh5c2lvdGhlcmFwaWUlMjBVTkQlMjBhbHRlcg==.

